# The Impact of Glucagon-like Peptide-1 (GLP-1) Receptor Agonists on Body Composition in Individuals with Overweight and Obesity: A Systematic Review and Meta-Analysis

**DOI:** 10.3390/jcm15176818

**Published:** 2026-09-03

**Authors:** Miłosz Woźniak, Zofia Tarcz, Gabriela Pyczek, Julia Bogacka, Andrzej Diniejko, Alina Kuryłowicz, Artur Mamcarz, Daniel Śliż

**Affiliations:** 1 Student Scientific Club of Lifestyle Medicine, 3rd Department of Internal Medicine and Cardiology, Medical University of Warsaw, 04-749 Warsaw, Poland; s088524@student.wum.edu.pl (M.W.); s088399@student.wum.edu.pl (G.P.); s088064@student.wum.edu.pl (J.B.); andrzej.diniejko@gmail.com (A.D.); 2 Department of Internal Medicine and Geriatric Cardiology, Centre of Postgraduate Medical Education, 00-416 Warsaw, Poland; alina.kurylowicz@cmkp.edu.pl; 3 3rd Department of Internal Medicine and Cardiology, Medical University of Warsaw, 04-749 Warsaw, Poland; artur.mamcarz@wum.edu.pl (A.M.); daniel.sliz@wum.edu.pl (D.Ś.)

**Keywords:** body composition, obesity, GLP-1 receptor agonists

## Abstract

**Background/Objectives**: Excess adipose tissue is associated with adverse changes in muscle metabolism and body composition. GLP-1 receptor agonists (GLP-1 RA) have transformed the treatment of obesity; however, concerns remain regarding the potential impact of treatment-associated weight loss on lean mass. This study aimed to evaluate the effects of GLP-1 RA on body composition in adults with obesity without type II diabetes mellitus (T2DM), with a particular focus on changes in lean mass. **Methods**: A database search of Medline Ultimate, Scopus, Web of Science, PubMed, and Embase was conducted to identify studies published up to December 30, 2024. Randomized controlled trials involving adults with obesity and without T2DM who received GLP-1 RA and reported changes in body composition were included. The protocol was registered with PROSPERO (CRD42025645378). Random-effects meta-analysis was performed using inverse-variance weighting. **Results**: A total of 2776 articles were identified, of which three trials comprising 171 participants met the inclusion criteria. Compared with controls, participants receiving GLP-1 RA treatment experienced greater reductions in lean mass (MD = −0.78 kg, 95% CI: [−1.37 to −0.16], I^2^ = 63.2%). GLP-1 RA treatment was also associated with a significant reduction in fat mass (MD = −3.43 kg, 95% CI: [−5.94 to −0.93], I^2^ = 78.2%). **Conclusions**: The loss of lean mass was greater in patients treated with GLP-1 RA than in the control group. These results highlight the importance for clinicians to incorporate lifestyle interventions, including physical activity and nutritional support, alongside therapy with GLP-1 RA.

## 1. Introduction

Obesity is a common worldwide disorder in which adverse changes occur in the structure and function of muscle tissue [1]. In obesity, adipose tissue, particularly the visceral depot, secretes adipokines and pro-inflammatory mediators that regulate muscle metabolism through endocrine and paracrine signaling [2,3]. These alterations, combined with physical inactivity, may contribute to sarcopenic obesity, a condition characterized by the coexistence of excess adiposity and impaired muscle health [4,5]. The reduced muscle-to-fat ratio complicates fat loss interventions and treatment [6,7]. Therefore, optimizing obesity management requires reducing fat mass while preserving muscle mass, for which physical activity is also beneficial.

Obesity is becoming a serious global health burden, and its prevalence is projected to rise substantially to 1.13 billion in the coming decade [8]. The introduction of GLP-1 RA has revolutionized the treatment of obesity. These agents produce substantial weight loss and improve cardiometabolic outcomes; however, their effects on body composition remain incompletely understood. This meta-analysis differs from prior reviews by analyzing articles describing the effect of GLP-1 RA on body mass components, while excluding trials involving confounding variables (medications, comorbidities) to isolate the direct impact of GLP-1 RA. This approach was chosen to evaluate the independent effect of GLP-1 RA on body composition while minimizing the influence of conditions and treatments known to alter lean and fat mass. By focusing on a more homogeneous population, the present review complements previous broader meta-analyses and provides evidence specifically applicable to adults with overweight or obesity without T2DM.

Previous studies have reported conflicting findings regarding the effects of GLP-1 RA on muscle-related outcomes. While some studies suggest reductions in muscle mass during treatment, others report preservation or improvement of muscle-related parameters [9,10,11].

The primary objective of this study was to evaluate the effects of GLP-1 RA therapy on body composition in adults with overweight or obesity without T2DM, with particular emphasis on lean mass. To minimize potential confounding, studies involving conditions or medications known to affect body composition were excluded. Improved understanding of these effects may help clinicians optimize obesity treatment strategies and guide efforts to preserve lean mass during weight reduction. This study aimed to evaluate whether GLP-1 RA therapy is associated with greater reductions in both lean mass and fat mass compared with control interventions and assess the importance of monitoring body composition and promoting lifestyle interventions during treatment.

## 2. Materials and Methods

The protocol for this systematic review was prospectively registered in the PROSPERO international prospective register of systematic reviews (CRD42025645378). This study followed the Preferred Reporting Items for Systematic Reviews and Meta-Analyses (PRISMA) 2020 statement [12]. No amendments were made to the registered protocol after registration.

### 2.1. Search Strategy and Selection Criteria

Only randomized controlled trials were included in this meta-analysis. On 30 December 2024, a predefined search strategy was applied without filters (Table S1). The search identified 4855 records from Embase, 521 from Medline Ultimate, 1643 from PubMed, 3512 from Scopus, and 1753 from Web of Science. Two reviewers contacted study authors to request missing data and obtain additional relevant information where necessary.

Eligible randomized controlled trials enrolled adults (≥18 years) with overweight or obesity and without T2DM who received GLP-1 RA and had a follow-up duration of at least 12 weeks. Interventions included GLP-1 RA monotherapy or GLP-1 RA added to a non-randomized background treatment unrelated to weight loss. Control groups consisted of participants receiving placebo or comparator interventions reported in peer-reviewed randomized controlled trials. Trials involving bariatric surgery or participants receiving weight-loss medications were excluded. Exclusion criteria were designed to minimize factors that could potentially influence body composition outcomes. Only articles published in English were considered. Consequently, several studies included in previous meta-analyses were excluded because they enrolled participants with T2DM, concomitant pharmacological weight-loss therapies, or comorbid conditions that could independently influence body composition.

Eligible studies reported either baseline and endpoint values or changes from baseline for body composition parameters, including body weight (kg), BMI (kg/m^2^), muscle mass (kg or %), lean mass (kg or %), fat mass (kg or %), visceral adipose tissue, subcutaneous adipose tissue, and waist circumference (cm). Only studies using dual-energy X-ray absorptiometry (DXA) to assess lean mass were included because of its accuracy, precision, and ability to provide regional body composition analysis.

Endpoint lean body mass and fat mass were obtained by subtracting change in lean/fat mass from baseline lean/fat mass. Endpoint mean body weight was measured by subtracting change in mean body weight from baseline mean body weight. Endpoint BMI in one study was obtained by subtracting change in BMI from baseline BMI [13]. In one study, endpoint visceral adipose tissue was estimated by subtracting the change value from the baseline value [14]. Endpoint subcutaneous adipose tissue was obtained by subtracting change value from baseline value [13,14]. Endpoint waist circumference was measured by subtracting the change value from the baseline value.

### 2.2. Data Analysis

After EndNote automatically removed 9251 duplicates, at least two independent researchers in each of the two groups (four researchers in total) screened records against the predefined exclusion criteria and identified any remaining duplicates. Study selection was conducted in two stages: screening of titles and abstracts, followed by full-text review. For studies meeting all inclusion criteria and none of the exclusion criteria, data were independently extracted by two researchers. Disagreements at any stage were resolved through discussion with a third reviewer until consensus was reached. No automated tools other than EndNote were used. Risk of bias was independently assessed by two reviewers using the Cochrane Risk of Bias 2 (RoB 2) tool (Figure S1). Any disagreements were resolved through discussion with a third reviewer until consensus was reached. No automation tools were used for the risk-of-bias assessment. Ultimately, three eligible studies were included in the meta-analysis. Studies were included in each quantitative synthesis according to the availability of outcome-specific data. Separate meta-analyses were performed for each outcome using all eligible studies that reported the corresponding outcome measure.

### 2.3. Statistical Methods

The effect measure for all continuous outcomes was the mean difference (MD) with corresponding 95% confidence intervals (95% CI). Outcomes included lean mass, fat mass, waist circumference, visceral adipose tissue, subcutaneous adipose tissue, and percentage changes in lean and fat mass, as appropriate. Within-study variances were computed from reported standard deviations (SD) and group sizes:

vi = (SD_1_^2^/n_1_) + (SD_2_^2^/n_2_).

When SDs were not available, they were derived from SE (SD = SE × √n) or from 95% confidence intervals (SE = (CI_high_ − CI_low_)/(2 × 1.96)).

Studies were combined using inverse-variance weighting under a random-effects model with restricted maximum likelihood (REML) estimation. Between-study heterogeneity was assessed with Cochran’s Q, the between-study variance (τ^2^; REML), and I^2^ (the percentage of total variability due to heterogeneity).

Primary synthesis was performed using a random-effects model with restricted maximum likelihood (REML) estimation. Sensitivity analyses were conducted using a leave-one-out approach, in which each comparison was sequentially omitted and the meta-analysis repeated. Additional influence diagnostics and Baujat plot analyses were performed to identify studies contributing disproportionately to heterogeneity and the pooled treatment effect.

Analyses were conducted in R version 4.5.0 (R Foundation for Statistical Computing) using the metafor (version 4.8.0) and meta (version 8.1.0) packages. Data preparation was performed using the readxl (version 1.4.5) and dplyr (version 1.1.4) packages.

Assessment of reporting bias (e.g., publication bias) was not performed because only three studies were included in the meta-analysis, making funnel plots and formal statistical tests unreliable.

Individual study results and pooled effect estimates were presented using forest plots. Study selection was summarized using a PRISMA flow diagram, and characteristics of included studies were summarized in tables and supplementary figures, as appropriate.

The certainty of the evidence for each outcome was assessed using the GRADE (Grading of Recommendations Assessment, Development and Evaluation) approach with GRADEpro GDT software (2023 version) (Figure S2) [15].

## 3. Results

**Figure 1:**
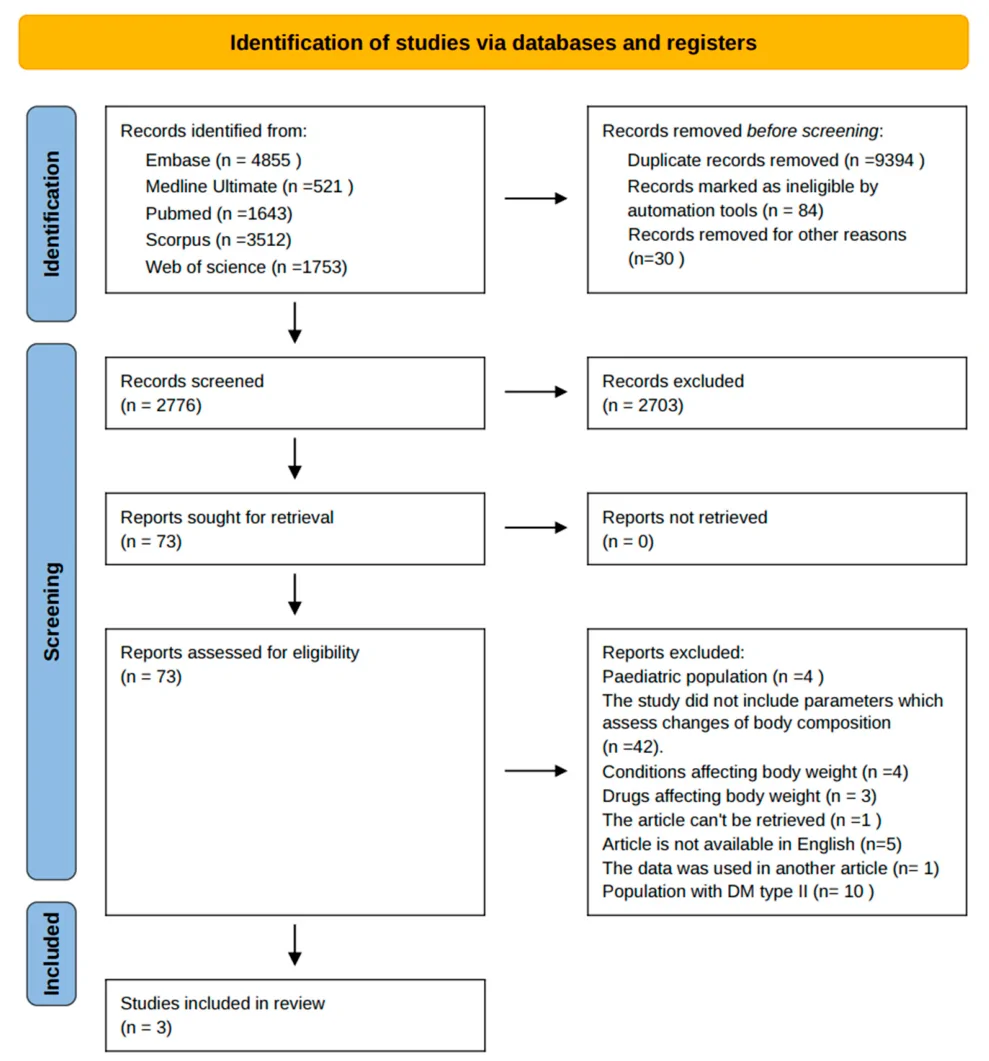
Flow chart. PRISMA flow diagram of the study selection process.

A total of 2776 articles were screened, of which 3 trials comprising 171 participants were included in the meta-analysis (Figure 1). Baseline characteristics of the included trials are presented in Table S2.

Assessment using GRADEpro GDT (2023 version) indicated moderate certainty of evidence for lean mass, fat mass, and waist circumference outcomes, and low certainty for subcutaneous fat mass and visceral fat mass (Figure S2).

### 3.1. Lean Mass

**Figure 2:**
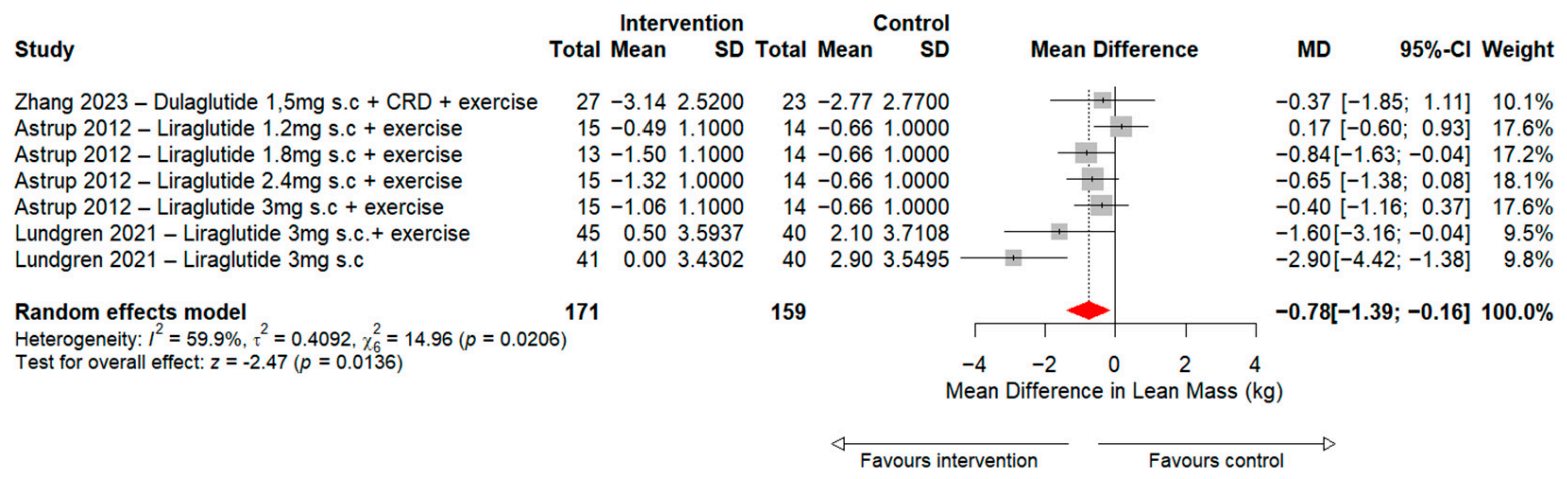
Forest plot of the mean difference in lean mass (kg) for GLP-1 RAs versus control [13,14,16].

**Figure 3:**
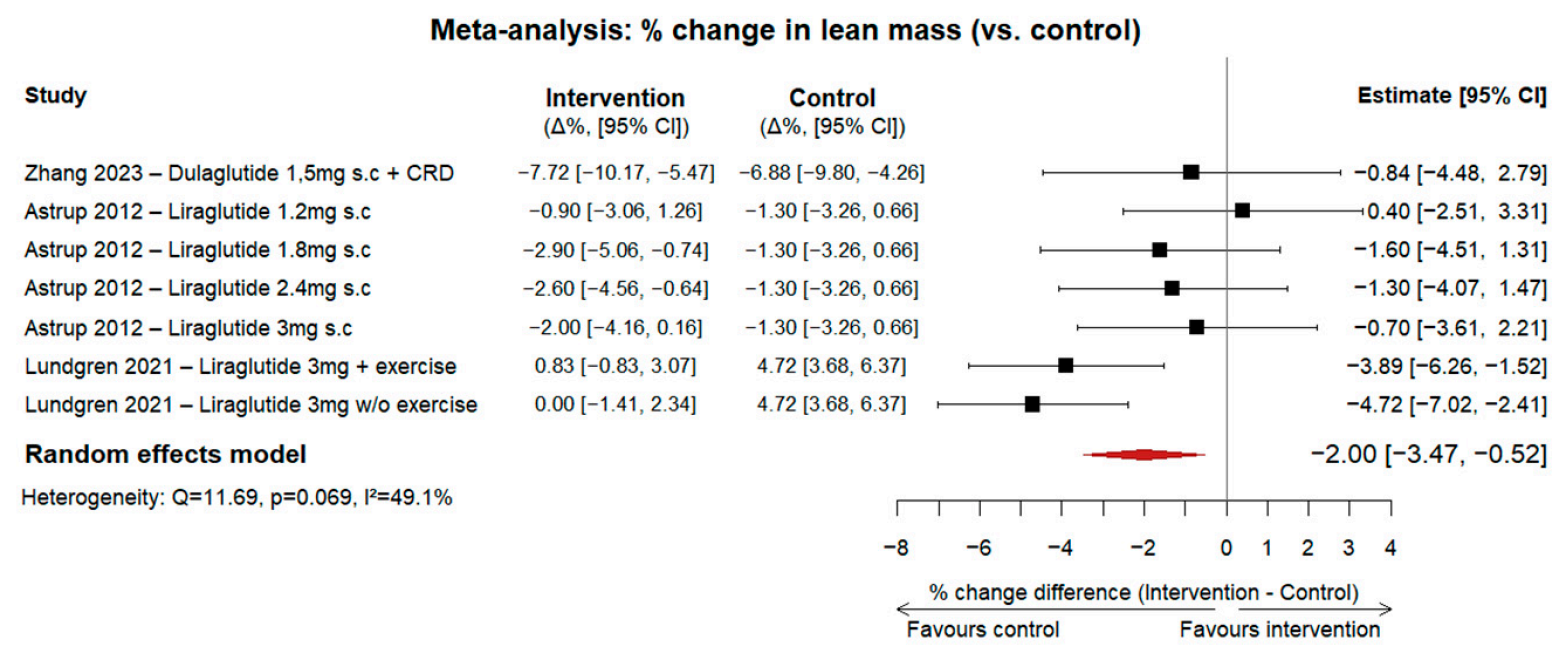
Forest plot of the percentage change in lean mass for GLP1-RAs versus control from meta-analysis [13,14,16].

Compared with the controls, greater reductions in DXA-derived lean mass, which includes non-muscular components, were observed in patients receiving GLP-1 RA treatment (MD = −0.78 kg; 95% CI: [−1.39 to −0.16]) (Figure 2). The pooled analysis showed that treatment with GLP-1 RA was generally associated with a modest decrease in the percentage of lean body mass relative to baseline. The estimated change was −2.00 percentage points (95% CI: [−3.47 to −0.52]) (Figure 3).

Sensitivity analyses supported the robustness of the primary lean mass meta-analysis. Leave-one-out analysis demonstrated that the pooled effect remained negative and statistically significant after sequential exclusion of each individual comparison, with pooled mean differences ranging from −0.49 to −0.94 kg (all *p* < 0.05). Although the magnitude of the effect varied modestly, no single comparison altered the direction or statistical significance of the overall finding. However, the Lundgren et al. liraglutide 3 mg without exercise comparison exerted a substantial influence on between-study heterogeneity [16]. Exclusion of this comparison reduced heterogeneity markedly (I^2^ = 12.8%, τ^2^ = 0.031), while the pooled effect remained statistically significant (MD = −0.49 kg, 95% CI −0.89 to −0.10). Consistent findings were obtained from influence diagnostics and the Baujat plot, which identified the same comparison as the principal contributor to heterogeneity owing to its divergent effect estimate rather than its statistical weight. Overall, these analyses indicate that the evidence for reduced lean mass was robust, although the precise magnitude of the effect was partly influenced by this single comparison. 

### 3.2. Fat Mass

**Figure 4:**
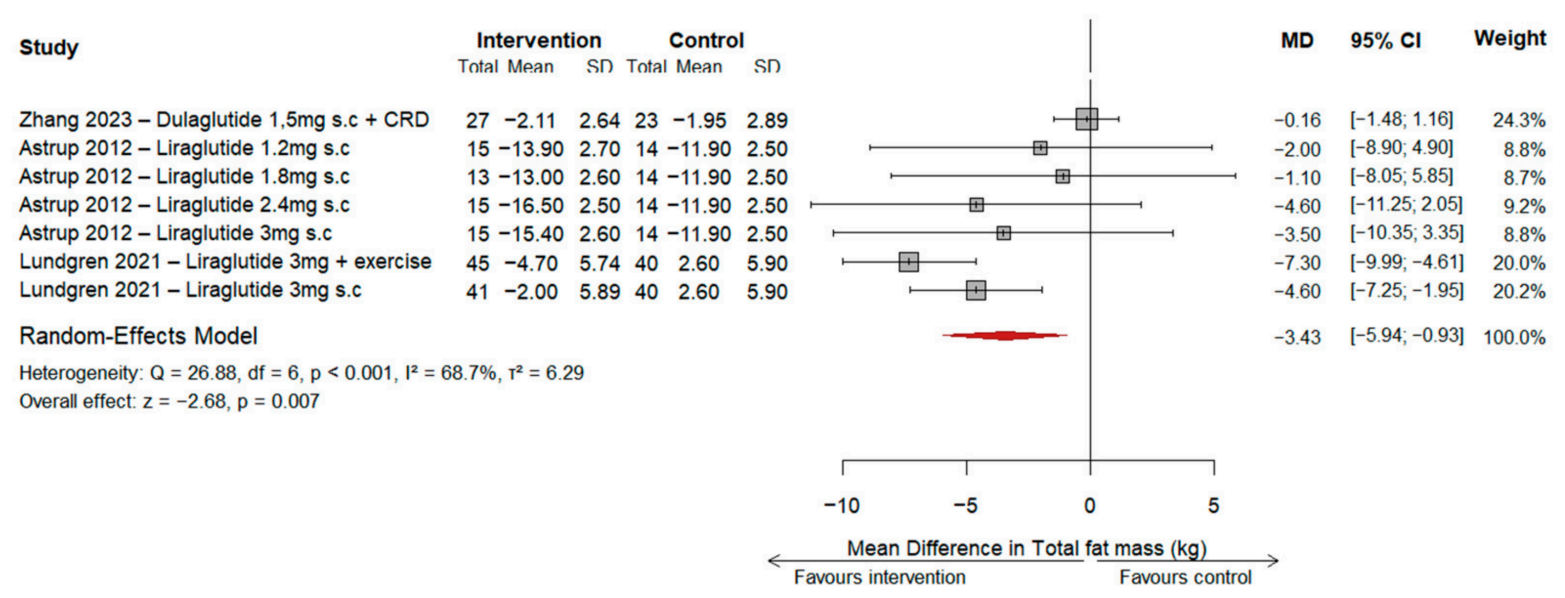
Forest plot of the mean difference in total fat mass (kg) for GLP-1 RA versus control [13,14,16].

**Figure 5:**
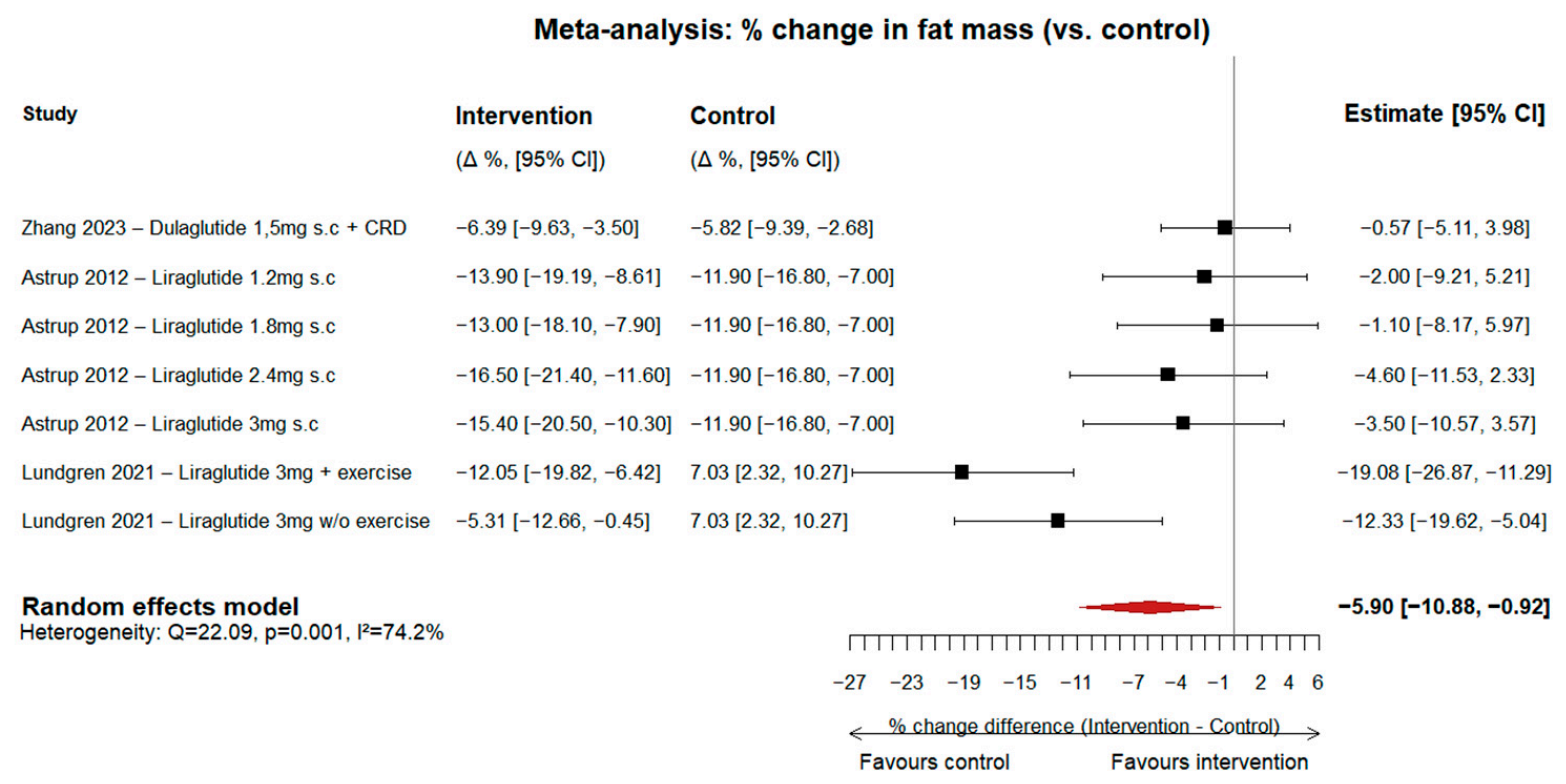
Forest plot of the percentage change in fat mass for GLP-1 receptor agonists versus control from meta-analysis [13,14,16].

Treatment with GLP-1 RA was associated with a significant reduction in fat mass. The MD was −3.43 kg (95% CI: [−5.94 to −0.93]) (Figure 4). The pooled analysis demonstrated that treatment with GLP-1 RA was consistently associated with a robust reduction in the percentage of fat mass relative to baseline. The mean estimated change was −5.90 percentage points (95% CI: [−10.88 to −0.92]) (Figure 5). Changes in fat mass and lean mass reported in the individual studies are presented in Figure S3.

### 3.3. Waist Circumference, Subcutaneous Fat Mass, and Visceral Fat Mass

The random-effects meta-analysis found that GLP-1 RA had no significant effect on visceral or subcutaneous fat mass. The overall MD in subcutaneous fat mass between the GLP-1 RA and control groups was −1.97 cm^2^ (95% CI: [−5.06 to 1.12]) (Figure S4), whereas the MD in visceral fat mass was −5.15 cm^2^ (95% CI: [−11.98 to 1.67]) (Figure S5). The GLP-1 RA group showed an MD in waist circumference of −3.65 cm (95% CI: [−5.52 to −1.77]) (Figure S6).

## 4. Discussion

The studies included in the meta-analysis analyzed the effect of treatment with GLP-1 RA on patients with overweight or obesity. Findings showed a decrease of 0.78 kg in lean mass in the intervention group compared to the control group (Figure 2). There was a mean 2.00% loss relative to baseline (Figure 3). The intervention group lost, on average, 3.43 kg more fat mass compared to the control group (Figure 4). Additionally, GLP-1 RA treatment was associated with a reduction in waist circumference (Figure S6). Jiao et al. found a reduction in lean mass of 1.02 kg compared to control [17]. Similarly, a recent study by Karakasis et al. found that GLP-1 RA reduced lean mass by an average of 0.86 kg compared to control, representing approximately 25% of the weight loss [18]. A systematic review by Bikou et al. exploring the impact of semaglutide on lean mass concluded that lean mass represents 0–40% of total weight reduction [19]. Methods of body composition assessment in these studies were varied, including DXA, MRI, and CT, which could have led to inconsistencies in measurements. Moreover, these studies included patients with variables that could influence outcomes, such as T2DM and drugs with known effects on body weight, which explains the differences in results. Therefore, rather than providing contradictory evidence, the present review complements previous meta-analyses by evaluating a more clinically homogeneous population in whom the isolated effects of GLP-1 RA can be assessed with fewer confounding influences.

Lean mass includes all components of the body that are not fat mass, such as bone mass, muscle mass, and organ tissue. A loss in lean mass cannot directly be attributed to any of the components. However, studies exploring the short-term effects of GLP-1 RA on bone metabolism, such as those by Anastasilakis et al. and Li et al., do not demonstrate a significant impact of incretin treatment on bone mineral density [20,21]. Nevertheless, caution must be applied when interpreting these studies in the context of obesity as their population encompasses mostly patients with T2DM. Consequently, the specific tissues contributing to reductions in lean mass remain uncertain. There is currently little research specifically exploring the effect of GLP-1 RA on muscle mass and muscle function. Existing research in this field focuses on the loss of lean mass. Landmark studies for GLP-1 RA, such as the STEP study by Garvey et al., lasting 104 weeks or the SCALE study from Wadden et al., spanning 56 weeks, did not collect data regarding muscle mass and bone mineral density [22,23].

According to EWGSOP2 guidelines, diagnosis of sarcopenia requires assessment of muscle quantity, muscle strength, and physical performance. The studies included in this review assessed body composition but did not systematically evaluate muscle strength or physical performance. Therefore, although the observed reduction in lean mass was modest, no conclusions regarding the presence or absence of sarcopenia can be drawn.

Strengths of this meta-analysis include the inclusion of randomized controlled trials. Additionally, two of the three studies were double blind [14,16]. One was an open label [13]. Body composition in all studies was measured using DXA (Dual-energy X-ray Absorptiometry). This method offers high accuracy and precision.

Limitations encompass the short duration and the small number of studies included, due to stringent exclusion and inclusion criteria. As a result, the number of patients analyzed is small. However, the group is free of factors influencing weight loss, such as conditions and drugs affecting body weight, which differentiates it from other meta-analyses. The small number of included studies and participants limits the generalizability of the findings and reduces the precision of the pooled estimates. By excluding studies involving concomitant pharmacological weight-loss therapies, the present review aimed to isolate the independent effects of GLP-1RA on body composition. Additionally, sample sizes in the studies were limited, and one study analyzed body composition parameters for only a subgroup of the study population [14]. In most studies, physical activity was advised (Table S2). One of the included studies analyzed the effect of dulaglutide on women with polycystic ovarian syndrome [13]. PCOS is characterized by metabolic disturbances, leading to insulin resistance, weight gain, accumulation of visceral adipose tissue and chronic inflammation, as reported by Yu et al. [24]. It is possible that the effect of a GLP-1 RA therapy in this group may differ from the effects in patients with just overweight or obesity. Clinical heterogeneity was present across included studies, including differences in GLP-1RA type, dose, treatment duration, exercise interventions, and participant characteristics. The Risk of Bias assessment, performed using the revised Cochrane risk-of-bias tool for randomized trials (RoB 2), did not reveal a significant risk of bias. Two studies ranked “Low Risk” [14,16] and one study ranked “Some Concerns” because of a “Some Concerns” rating in Domain 3: Risk of bias due to missing outcome data [13]. In this study, a substantial number of participants were lost to follow-up. Formal assessment of publication bias was not performed because only three studies were eligible for inclusion, making such analyses unreliable.

According to Rubino et al., discontinuation of GLP-1 RA therapy can lead to weight regain [25]. GLP-1 RA are now being considered for long-term use. Assessing the long-term impact of these therapies on body composition is not possible given that they are relatively new, and extended follow-up data is not available. The potential role of physical activity, particularly resistance exercise, in preserving lean tissue during GLP-1 RA treatment warrants further investigation. As GLP-1 RA decrease appetite, food intake will also be reduced. This may lead to deficiencies in protein, micronutrients, and vitamins. Fallows warns against the risk of malnutrition in patients taking GLP-1 RA and calls for an assessment of the patients’ nutritional status to ensure health outcomes [26]. It is also important to note that changes in lean mass could be more pronounced and have more severe implications in older patients, who are at risk of sarcopenia, according to Makizako [27].

Further studies are needed to determine the impacts of long-term therapy with GLP-1 RA on parameters such as lean mass, muscle mass, muscle function, and bone mineral density. Care should also be applied to study these parameters in different populations, paying attention to changes in the elderly population and in women, where fat distribution and metabolism may differ.

## 5. Conclusions

This systematic review and meta-analysis evaluated changes in body composition associated with GLP-1 RA therapy in adults with overweight or obesity without T2DM. Treatment with GLP-1 RA was associated with greater reductions in both fat mass and lean mass compared with control interventions, with fat mass accounting for most of the observed tissue loss. These findings highlight the importance of monitoring body composition during obesity treatment rather than focusing exclusively on changes in body weight. However, these findings are based on only three randomized controlled trials and should therefore be interpreted with caution. Given the potential clinical relevance of lean mass preservation, clinicians should encourage adequate nutritional intake and regular physical activity, particularly resistance exercise, alongside pharmacological treatment. Further research is needed to determine the long-term effects of GLP-1 RA therapy on body composition and to evaluate outcomes that were not systematically assessed in the available studies, including muscle strength and physical performance.

## Data Availability

The data analyzed in this study were derived from publicly available studies cited in the reference list. No additional datasets, analytic code, or other review materials are publicly available.

## Supplementary Materials

The following supporting information can be downloaded at: https://www.mdpi.com/article/10.3390/jcm15176818/s1, Table S1. Literature Search Strategy; Table S2. Baseline Characteristics of Included Studies; Figure S1. Risk of Bias Summary; Figure S2. GRADE Assessment; Figure S3. Changes in Fat Mass and Lean Mass in Individual Studies (kg); Figure S4. Forest Plot of the Mean Difference in Subcutaneous Adipose Tissue for GLP-1 Receptor Agonists versus Control; Figure S5. Forest Plot of the Mean Difference in Visceral Adipose Tissue for GLP-1 Receptor Agonists versus Control; Figure S6. Forest Plot of the Mean Difference in Waist Circumference for GLP-1 Receptor Agonists versus Control.
