# Supplementary material for: The Impact of Glucagon-like Peptide-1 (GLP-1) Receptor Agonists on Body Composition in Individuals with Overweight and Obesity: A Systematic Review and Meta-Analysis
Source: J Clin Med. 2026 Sep 3;15(17):6818. doi: 10.3390/jcm15176818 (PMC13566191; doi:10.3390/jcm15176818)
Supplement: Supplementary file 1 [file jcm-15-06818-s001.zip › jcm-4469105-supplementary.pdf]

The Impact of Glucagon-Like Peptide-1 (GLP-1) Receptor Agonists on Body Composition in Individuals with Overweight and Obesity: A systematic review and meta-analysis

Authors and affiliations:

Miłosz Woźniak[1], Zofia Tarcz[1], Gabriela Pyczek[1], Julia Bogacka[1], Andrzej Diniejko[1], Alina Kuryłowicz[2], Artur Mamcarz[3], Daniel Śliż[3]

[1] Student Scientific Club of Lifestyle Medicine at 3rd Department of Internal Medicine and Cardiology, Medical University of Warsaw, Warszawa, Poland

[2] Department of Internal Medicine and Geriatric Cardiology, Centre of Postgraduate Medical Education, Warsaw, Poland

[3] 3rd Department of Internal Medicine and Cardiology, Medical University of Warsaw, Warszawa, Poland

Details of the corresponding author:

Zofia Tarcz

s088484@student.wum.edu.pl

Medical University of Warsaw

Banacha 1A, 02-097 Warsaw, Poland

Tel.: +48 572 506 226

Statement and Declarations:

Conflict of interest: None declared.

Acknowledgments: None declared.

Key words: body composition, obesity, GLP-1 receptor agonists, sarcopenia

Supplementary Materials

Table of content

Table S1: Literature Search Strategy

Table S2: Initial parameters: Baseline Characteristic of Included Studies

Figure S1: Risk of Bias – Summary

Figure S2: GRADE
Figure S3: Change in fat mass and lean mass in the individual studies (kg).

Figure S4: Forest plot of the mean difference in subcutaneous adipose tissue (cm^2) for GLP-1 receptor agonists versus control.

Figure S5: Forest plot of the mean difference in visceral adipose tissue (cm^2) for GLP-1 receptor agonists versus control.

Figure S6: Forest plot of the mean difference in waist circumference (cm) for GLP-1 receptor agonists versus control.

Table S1: Literature Search Strategy

EMBASE

1. (liraglutide OR Victoza OR Wegovy OR semaglutide OR Ozempic OR Rybelsus OR dulaglutide OR Trulicity OR lixisenatide OR exenatide OR Byetta OR Bydureon OR Saxenda OR tirzepatide OR Mounjaro OR GLP-1 agonist OR GLP-1 receptor agonist OR GLP-1 RA OR glucagon-like peptide).tw.
2. (body composition OR fat mass OR fat mass percentage OR lean mass OR lean mass percentage OR muscle mass OR muscle mass loss OR muscle mass percentage OR lean body weight).tw.
3. 1 AND 2

Medline Ultimate

1. (liraglutide OR Victoza OR Wegovy OR semaglutide OR Ozempic OR Rybelsus OR dulaglutide OR Trulicity OR lixisenatide OR exenatide OR Byetta OR Bydureon OR Saxenda OR tirzepatide OR Mounjaro OR GLP-1 agonist OR GLP-1 receptor agonist OR GLP-1 RA OR glucagon-like peptide).tw.
2. (body composition OR fat mass OR fat mass percentage OR lean mass OR lean mass percentage OR muscle mass OR muscle mass loss OR muscle mass percentage OR lean body weight).tw.
3. 1 AND 2

PubMed

1. (liraglutide OR Victoza OR Wegovy OR semaglutide OR Ozempic OR Rybelsus OR dulaglutide OR Trulicity OR lixisenatide OR exenatide OR Byetta OR Bydureon OR Saxenda OR tirzepatide OR Mounjaro OR GLP-1 agonist OR GLP-1 receptor agonist OR GLP-1 RA OR glucagon-like peptide).tw.
2. (body composition OR fat mass OR fat mass percentage OR lean mass OR lean mass percentage OR muscle mass OR muscle mass loss OR muscle mass percentage OR lean body weight).tw.
3. 1 AND 2

Scopus

1. (liraglutide OR Victoza OR Wegovy OR semaglutide OR Ozempic OR Rybelsus OR dulaglutide OR Trulicity OR lixisenatide OR exenatide OR Byetta OR Bydureon OR Saxenda OR tirzepatide OR Mounjaro OR GLP-1 agonist OR GLP-1 receptor agonist OR GLP-1 RA OR glucagon-like peptide).tw.
2. (body composition OR fat mass OR fat mass percentage OR lean mass OR lean mass percentage OR muscle mass OR muscle mass loss OR muscle mass percentage OR lean body weight).tw.
3. 1 AND 2

Web of Science

1. (liraglutide OR Victoza OR Wegovy OR semaglutide OR Ozempic OR Rybelsus OR dulaglutide OR Trulicity OR lixisenatide OR exenatide OR Byetta OR Bydureon OR Saxenda OR tirzepatide OR Mounjaro OR GLP-1 agonist OR GLP-1 receptor agonist OR GLP-1 RA OR glucagon-like peptide).tw.
2. (body composition OR fat mass OR fat mass percentage OR lean mass OR lean mass percentage OR muscle mass OR muscle mass loss OR muscle mass percentage OR lean body weight).tw.
3. 1 AND 2

Table S2: Initial parameters: Baseline Characteristic of Included Studies [12,13,21].

Author name and year	N	GLP-1 receptor agonists and dose	Duration (weeks)	Body weight (kg)	BMI (kg/m^2)	Fat mass (kg)	Lean mass (kg)	Exercise training – kind	Exercise training – monitoring of exercise	Female (%)
Zhang 2023	27	Dulaglutide 1•5mg s.c	24	77•62	29•68	33•03	40•68	Aerobic and resistance	Yes	100
Astrup 2012	15	Liraglutide 1•2mg s.c	20	96•20	34•8	43•50	55	Not specified	Not specified	77
	13	Liraglutide 1•8mg s.c		98•00	35•0	45•00	51•7			76
	15	Liraglutide 2.4mg s.c		98•40	35•0	42•60	50•6			76
	15	Liraglutide 3mg s.c		97•60	34•8	43•90	53•1			75
Lundgren 2021	41	Liraglutide 3mg s.c	52	95•10	32•7	39•3	58•9	No training	No	64
	45	Liraglutide 3mg s.c		98•30	32•8	39•5	60•5	Aerobic	Yes	64

Figure S1: Risk of Bias – Summary [12,13,21].

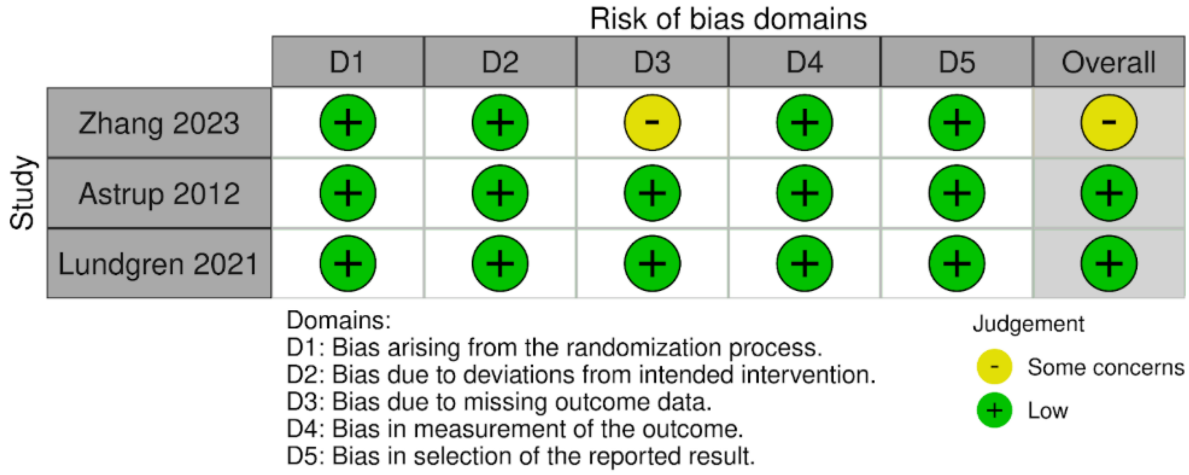

Figure S2: GRADE

Author(s): Miłosz Woźniak[1], Zofia Tarcz[1], Gabriela Pyczek[1], Julia Bogacka[1], Andrzej Diniejko[1], Alina Kuryłowicz[2] , Artur Mamcarz[3], Daniel Śliż[3]

Title: The Impact of Glucagon-Like Peptide-1 (GLP-1) Receptor Agonists on Body Composition in Individuals with Overweight and Obesity: A systematic review and meta-analysis

Certainty assessment							№ of patients		Effect		Certainty	Importance
№ of studies	Study design	Risk of bias	Inconsistency	Indirectness	Imprecision	Other considerations	analogs GLP-1	other drugs	Relative (95% CI)	Absolute (95% CI)		
**Mean difference in lean mass (kg)**												
3	randomised trials	not serious	serious[a]	not serious	not serious	none	171	159	-	MD **0.78kg lower** (1.39 lower to 0.16 higher)	⨁⨁⨁◯ Moderate[a]	
**Mean difference in fat mass (kg)**												
3	randomised trials	not serious	serious[a]	not serious	not serious	none	171	159	-	MD 3.**43 kg lower** (5.94 lower to 0.93 higher)	⨁⨁⨁◯ Moderate[a]	
**Mean difference in waist circumference (cm)**												
3	randomised trials	not serious	serious[a]	not serious	not serious	none	171	159	-	MD **3.65 cm lower** (5.52 lower to 1.77 higher)	⨁⨁⨁◯ Moderate[a]	
**Mean difference in subcutaneous fat mass (cm²)**												
2	randomised trials	not serious	not serious	not serious	very serious[b]	none	85	79	-	MD **1.97 kg lower** (5.06 lower to 1.12 higher)	⨁⨁◯◯ Low[b]	
**Mean difference in visceral fat mass (cm²)**												
2	randomised trials	not serious	not serious	not serious	very serious[b]	none	85	79	-	MD **5.15 kg lower** (11.98 lower to 1.67 higher)	⨁⨁◯◯ Low[b]	

CI: confidence interval; **MD:** mean difference

Explanations

a. Statistical analyses show significant heterogeneity.

b. The 95% confidence interval was relatively wide, indicating uncertainty regarding the magnitude of the effect, and the total sample size was limited.

Figure S3: Change in fat mass and lean mass in the individual studies (kg) [12,13,21].

Data presented as absolute changes in kilograms (kg) from baseline for each study arm. [1]BMI-Body Mass Index.

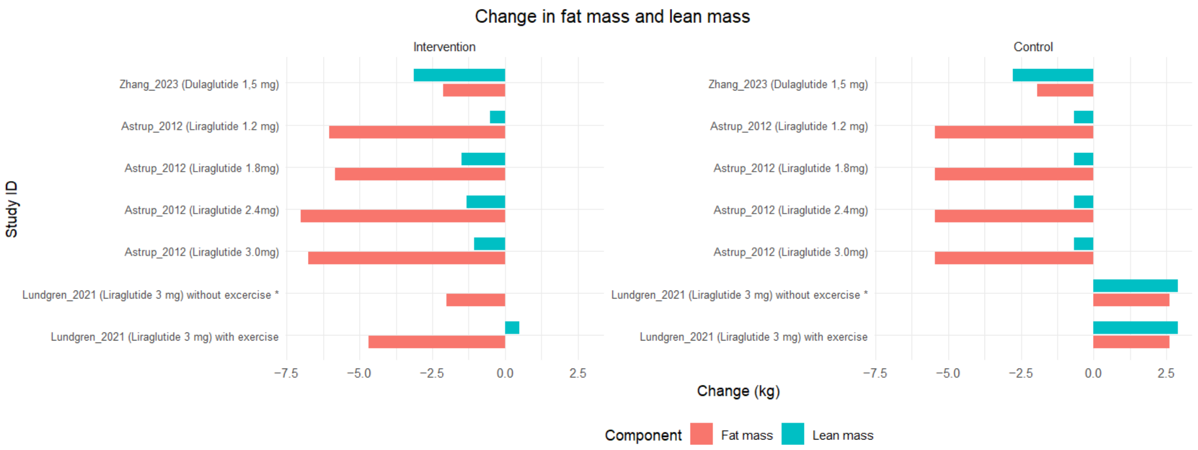

[1] BMI- Body Mass Index.

Figure S4: Forest plot of the mean difference in subcutaneous adipose tissue (cm^2) for GLP-1 receptor agonists versus control [12,13,21]..

Analysis conducted using random-effects model. [2]CRD- Calorie-Restricted Diet; s.c.- subcutaneous; CI-Confidence Interval.

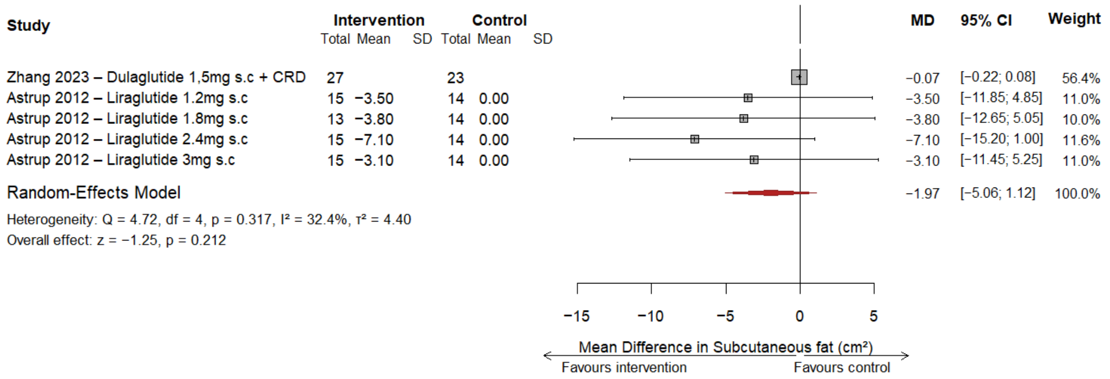

[2] CRD- Calorie-Restricted Diet; s.c.- subcutaneous; CI-Confidence Interval.

Figure S5: Forest plot of the mean difference in visceral adipose tissue (cm²) for GLP-1 receptor agonists versus control [12,13,21]..

Analysis conducted using random-effects model. [3]CRD- Calorie-Restricted Diet; s.c.- subcutaneous; CI-Confidence Interval.

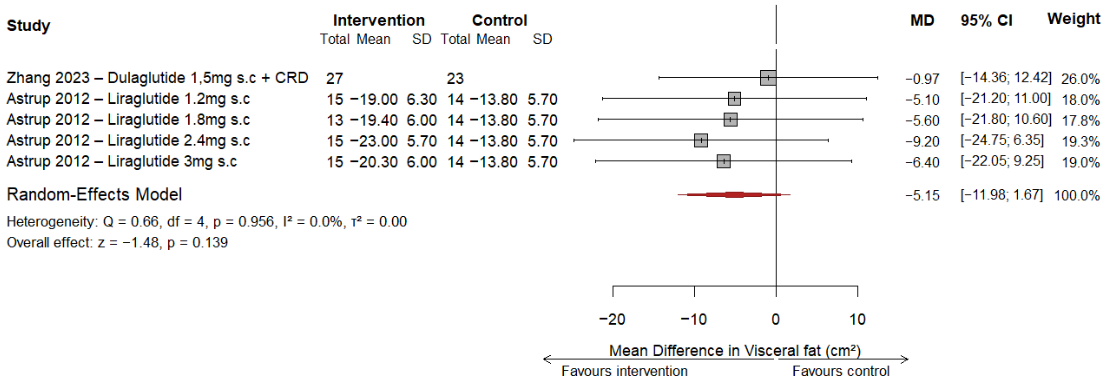

[3] CRD- Calorie-Restricted Diet; s.c.- subcutaneous; CI-Confidence Interval.

Figure S6: Forest plot of the mean difference in waist circumference (cm) for GLP-1 receptor agonists versus control [12,13,21].

Analysis conducted using random-effects model. [4]CRD- Calorie-Restricted Diet; s.c.- subcutaneous; CI-Confidence Interval.

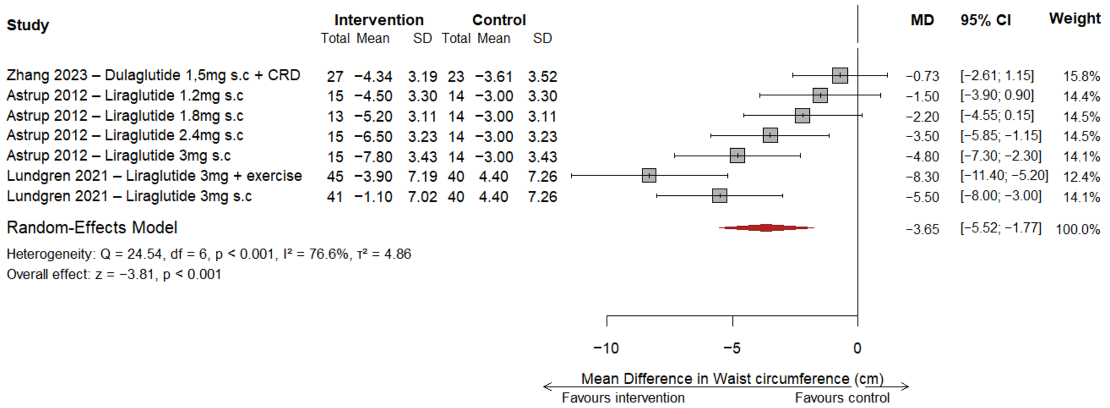

---

[4] CRD- Calorie-Restricted Diet; s.c.- subcutaneous; CI-Confidence Interval.
